# HIV interactions and the perils of epithelial thinning in the female reproductive tract

**DOI:** 10.1186/1742-4690-9-S2-P363

**Published:** 2012-09-13

**Authors:** A Carias, M McRaven, M Anderson, T Henning, E Kersh, J Smith, K Butler, S Vishwanathan, JM McNicholl, RM Hendry, R Veazey, T Hope

**Affiliations:** 1 Northwestern University, Chicago, IL, USA; 2 Center for Disease Control and Prevention, Atlanta, GA, USA; 3 Tulane National Primate Research Center, Covington, LA, USA

## Background

Currently, there is much debate on whether epithelial thinning from hormonal contraceptives can increase HIV acquisition. Previously, we illustrated that HIV can penetrate to depths in squamous epithelium where it can interact with target cells, such as CD4+ T-cells and macrophages. Using a similar approach, we show that epithelial thinning affects virus penetration, along with target cell and cellular junction distribution.

## Methods

To investigate how genital epithelial thickness may affect HIV penetration, ten female rhesus macaques were pre-treated with 30mg depo-medroxyprogesterone acetate (Depo-provera®) 4-5 weeks prior to vaginal photoactivatable (PA-GFP) HIV exposure. Additionally, eight female pigtail macaques were exposed to PA-GFP HIV at various menstrual cycle stages. Genital tracts were removed 4 hours post-exposure and immediately dissected and snap frozen in optimal cutting temperature (OCT) compound. Comparison of pre- and post-photoactivation image z-stacks revealed the presence of virus, accounting for background.

## Results

Within 4 hours, PA-GFP virions were observed between squamous epithelial cells penetrating up to depths of 50μm. This is within the reach of target cell populations. Furthermore, current analysis illustrated epithelial thickness to be inversely proportional to the number of penetrating virions and target cells, independent of thinning mechanism. Also, cellular junction distribution in pigtail macaques with thinned squamous epithelia mirrored those results of progesterone-treated rhesus macaques.

## Conclusion

Our current results suggest that HIV acquisition in women may be influenced by menstrual cycle and hormonal contraceptives. CD4+ T-cells and CD68+ macrophage distribution and virus penetration were dependent on epithelial thickness, suggesting HIV interactions with female genital epithelia may differ in the luteal and follicular stages of the menstrual cycle. Our results also suggest that progestin-based contraceptives may alter the barrier properties of the stratified squamous epithelium, possibly increasing the risk of HIV acquisition in women.

